# An unusually stable chlorophosphite: What makes BIFOP–Cl so robust against hydrolysis?

**DOI:** 10.3762/bjoc.11.36

**Published:** 2015-03-04

**Authors:** Roberto Blanco Trillo, Jörg M Neudörfl, Bernd Goldfuss

**Affiliations:** 1 Department for Chemistry, Institut für Organische Chemie, Universität zu Köln, Greinstr. 4, 50939 Köln, Germany

**Keywords:** chirality, hydrolysis, phosphorus, rearrangements, terpenoids

## Abstract

Two chlorophosphites, the biphenyl-based BIFOP–Cl and the diphenyl ether-based O–BIFOP–Cl, exhibit striking differences regarding their reaction with water. While BIFOP–Cl is nearly completely unreactive, its oxo-derivative O–BIFOP–Cl reacts instantly with water, yielding a tricyclic hydrocarbon unit after rearrangement. The analysis of the crystal structure of O–BIFOP–Cl and BIFOP–Cl revealed that the large steric demand of encapsulating fenchane units renders the phosphorus atom nearly inaccessible by nucleophilic reagents, but only for BIFOP–Cl. In addition to the steric effect, a hypervalent P(III)–O interaction as well as an electronic conjugation effect causes the high reactivity of O–BIFOP–Cl. A DFT study of the hydrolysis in BIFOP–Cl verifies a higher repulsive interaction to water and a decreased leaving tendency of the chloride nucleofuge, which is caused by the fenchane units. This high stability of BIFOP–Cl against nucleophiles supports its application as a chiral ligand, for example, in Pd catalysts.

## Introduction

**Scheme 1:**
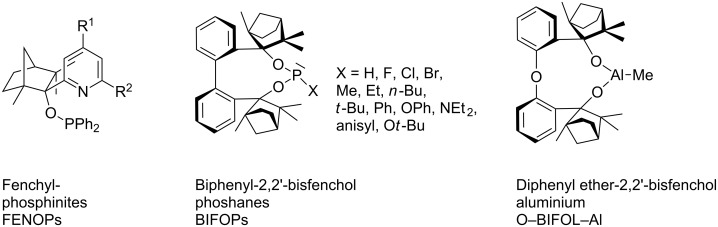
Fenchyl-based ligands used as building blocks for phosphorous ligands or organoaluminum reagents.

Phosphorus halides are highly reactive intermediates for the synthesis of phosphites and phosphoramidites [1–5], which are widely used, for example, as ligands in catalysts [6–9]. There are also some applications of phosphine halides used as ligands in catalytic reactions, for example, in cross-coupling reactions and hydroformylations [10–12]. We recently reported the application of the fenchole-based, phosphine halide BIFOP–Hal (Hal = F, Cl, Br) (Scheme 1) in an intramolecular palladium-catalyzed alkyl–aryl cross-coupling reaction [13] and in Pd-catalyzed allylic substitutions [14]. Several of the highly sterically hindered BIFOP derivatives were employed as ligands in Cu-catalyzed 1,4-additions [15]. Similar chelating fencholates [16–22] (Scheme 1) were employed in enantioselective organozinc catalysis reactions [23–26], umpolung catalysis [27] and in organoaluminum [17] and chiral *n*-butyllithium aggregates [28–33].

**Scheme 2:**
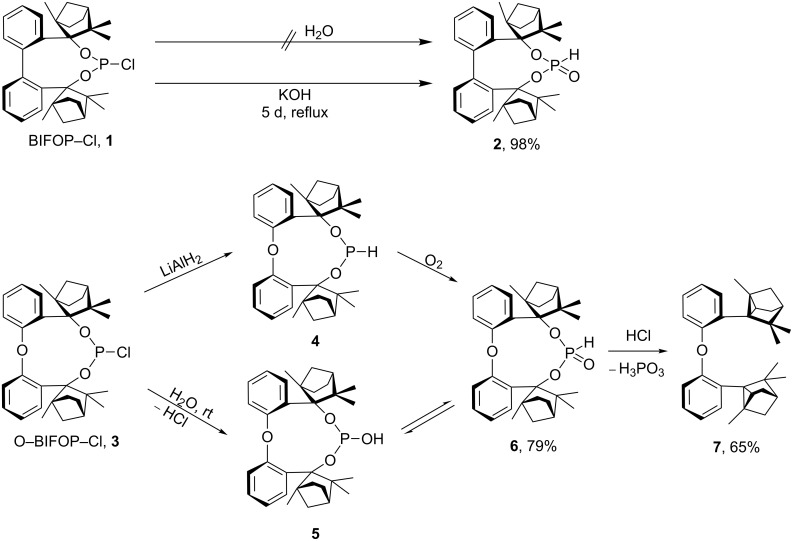
Reaction of BIFOP–Cl (**1**) to BIFOP–(O)H (**2**) and of O–BIFOP–Cl (**3**) yielding O–BIFOP–H (**4**), O–BIFOP–(O)H (**6**) as well as diphenyl ether-2,2’-biscyclofenchene **7**.

The chlorophosphite BIFOP-Cl (**1**) is air-stable and very resistant to hydrolysis (Scheme 2) [13,15]. The low reactivity of **1** to O- and C-nucleophiles is explained by the tight encapsulation of the P–Cl unit of the *endo*-fenchane moieties [15]. This unusual stability of the BIFOP–halides prompted the comparison of BIFOP–Cl (**1**) with its diphenyl ether derivative O–BIFOP–Cl (**3**). Despite similar encapsulation by two fencholate moieties, O–BIFOP–Cl **3** exhibits a significantly higher reactivity with nucleophiles (e.g., with water). Here we rationalize the different reactivities of **1** and **3**.

## Results and Discussion

**Figure 1:**
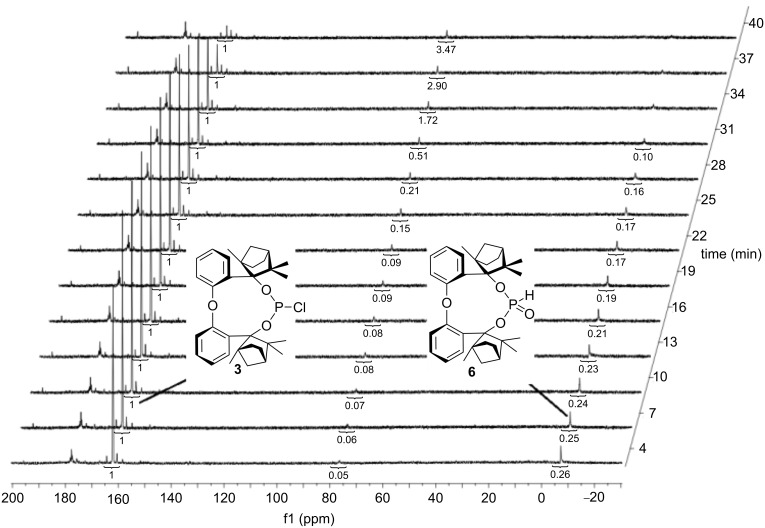
^31^P NMR (125 MHz, CDCl_3_) of O–BIFOP–Cl (**3**, δ = 161.9) after the addition of 1 equiv H_2_O and formation of O–BIFOP–(O)H (**6**, δ = −8.2), which vanished after 37 min forming **7** (cf. Scheme 2 and Scheme 3). The integration values are shown below the signals.

**Figure 2:**
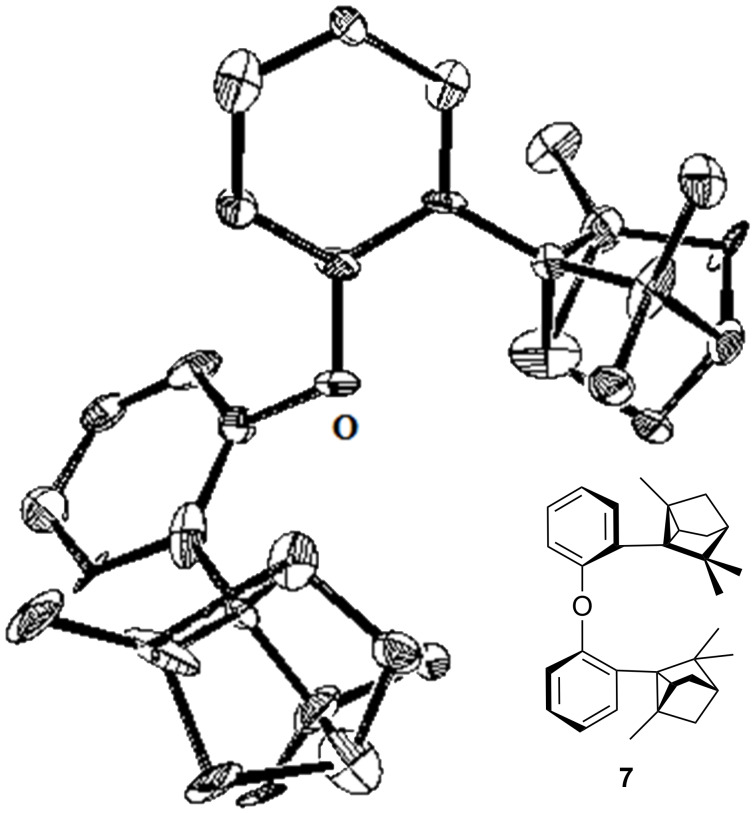
X-ray crystal structure of diphenyl ether-2,2’-biscyclofenchene **7**. Ellipsoids are shown with 50% probability.

In contrast to BIFOP–Cl (**1**), the diphenyl ether analogue O–BIFOP–Cl (**3**) exhibits the expected halophosphite reactivity and instantly reacts with water (Scheme 2, Figure 1). NMR monitoring of the hydrolysis showed that O–BIFOP–Cl (**3**, ^31^P NMR, δ = 161.9, Figure 1) is immediately hydrolyzed, yielding O–BIFOP–(O)H (**6**, ^31^P NMR, δ = −8.2, Figure 1). After 37 min, the amount of starting O–BIFOP–Cl (**3**) as well as the primary hydrolysis product **6** (Figure 1) is nearly completely depleted. The details of the reaction mixture that yielded diphenyl ether-2,2’-biscyclofenchene **7** (Figure 2) are shown in Scheme 2.

**Scheme 3:**
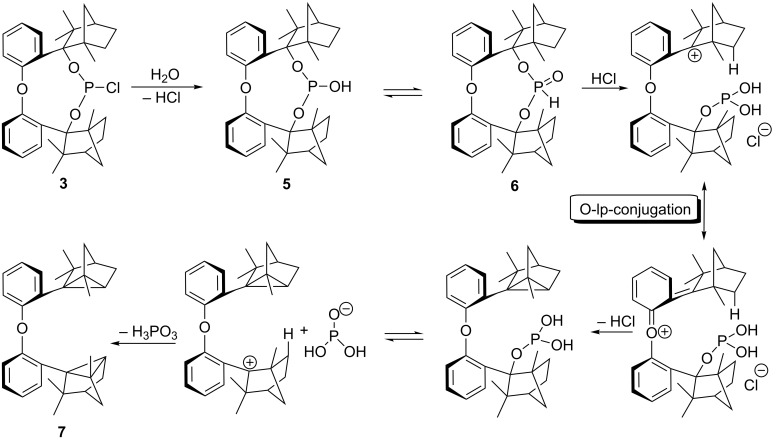
Proposed mechanism for the formation of diphenyl ether-2,2’-biscyclofenchene **7** through stabilization of the intermediate carbocation by O-lp conjugation and cyclopropane formation starting from O–BIFOP–Cl (**3**).

The formation of a cyclopropane ring in **7** can be rationalized to proceed through a fenchyl carbocation (Scheme 3) [34–40]. Intramolecular cyclopropanation reactions are often characterized by prolonged treatment with an acid [41–46]. Stabilization of the intermediate carbocation by the lone pair of the oxygen atom is enabled by lone-pair conjugation (O-lp conjugation) of the benzyl cation and supports elimination of the oxido unit (Scheme 3).

**Figure 3:**
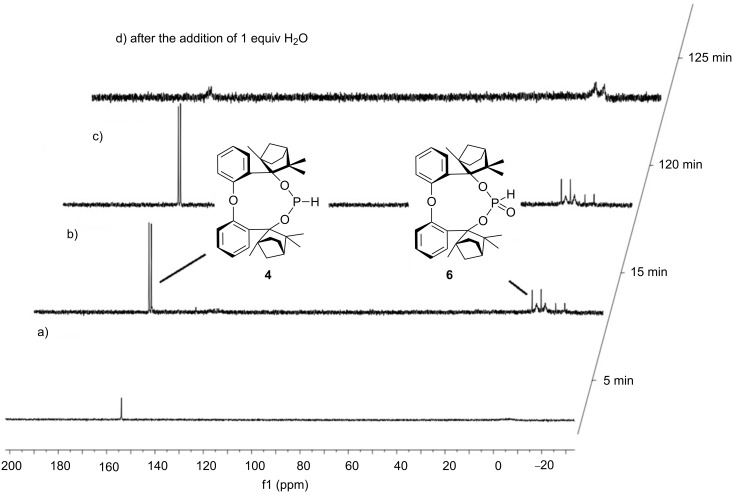
^31^P NMR (125 MHz, CDCl_3_) of O–BIFOP–H (**4**, δ = 152.5) adding O_2_ after a) 5 min; b) 15 min; c) 120 min; d) adding 1 equiv H_2_O forming O-BIFOP-(O)H (**6**, δ = −8.2).

To assess whether this rearrangement, formaing **7**, is mediated by HCl originating from **3**, chlorine-free O–BIFOP–H (**4**) was treated with O_2_, yielding **6** (79%, Scheme 2). While O–BIFOP–H (**4**) readily reacts with water, O–BIFOP–(O)H (**6**) was found to be stable in air and water (Figure 3d). However, addition of HCl to O–BIFOP–(O)H (**6**) gave diphenyl ether-2,2’-biscyclofenchene **7** in 65% yield. Hence, acidic conditions (HCl) are necessary to form **7** from **6**, which is generated by hydrolysis of 3 (Scheme 2 and Scheme 3).

**Figure 4:**
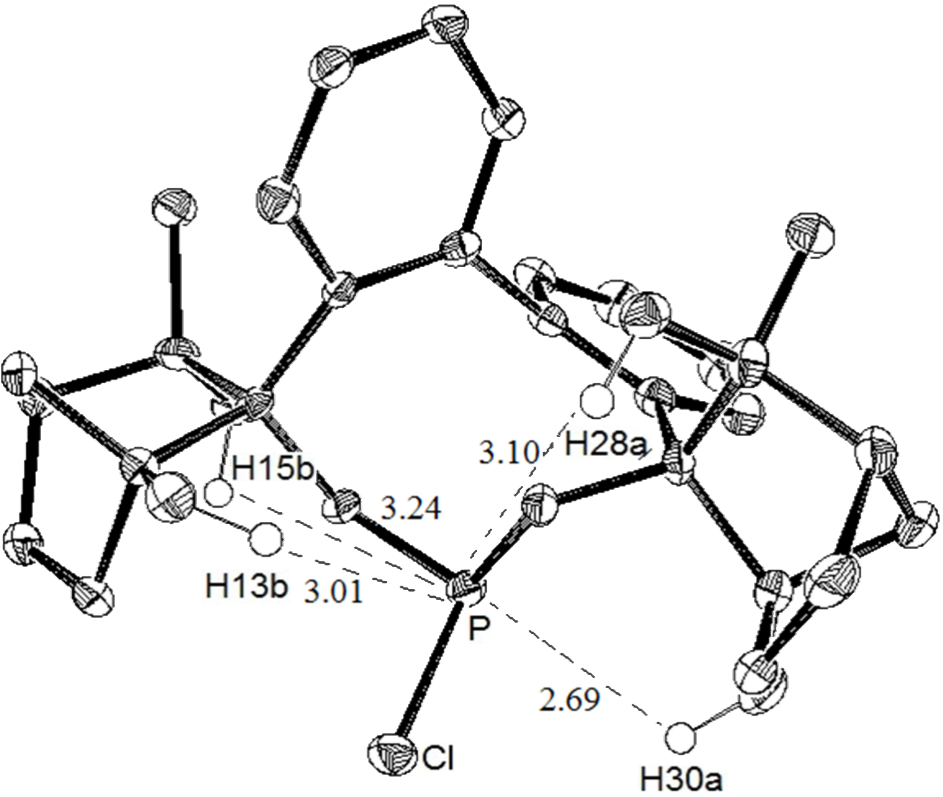
X-ray crystal structure of BIFOP-Cl **1**. Ellipsoids are shown with 50% probability [15].

**Scheme 4:**
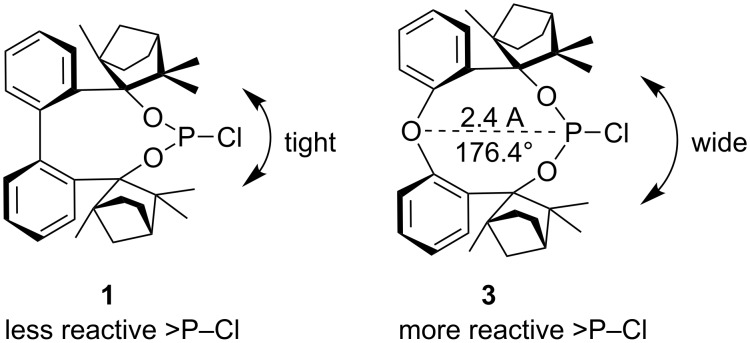
The different backbones provoke different reactivities due to tighter encapsulation of the P–Cl unit by the fenchane moieties in BIFOP–Cl (**1**) relative to O–BIFOP–Cl (**3**).

**Figure 5:**
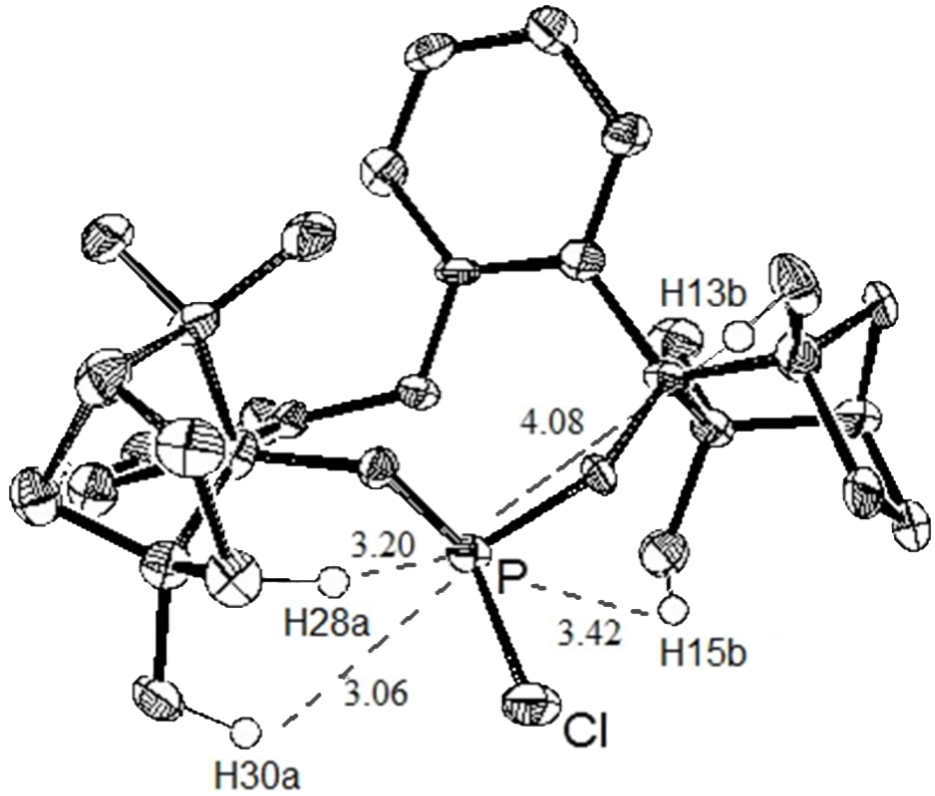
X-ray crystal structure of O-BIFOP-Cl (**3**). Ellipsoids are shown with 50% probability.

The analysis of the crystal structure of BIFOP–Cl (**1**) reveals the large steric demand of the fenchane units, which embed the phosphorus atom, thus making it inaccessible to nucleophilic reagents (Figure 4, Table 1) [14–15]. In contrast, the reduced protection of the phosphorus atom in O-BIFOP-Cl (**3**), which is primarily caused by the relatively large H13b–P distance (4.08 Å to 3.01 Å), provides an explanation for the higher reactivity of the >P–Cl moiety in O–BIFOP–Cl (**3**, Scheme 4, Figure 5, Table 1).

**Table 1:** Geometries bases on the X-ray structure of BIFOP–Cl^a^ and O–BIFOP–Cl.

	BIFOP–Cl (**1**)	O–BIFOP–Cl (**3**)

Angle sum at P (°)^b^	305.2	290.7
FAA–lp (°)^c^	38.9	−26,6
FAA (°)^c^	37.1	−52.5
H13b–P (Å)	3.01	4.08
H15b–P (Å)	3.24	3.42
H28a–P (Å)	3.10	3.20
H30a–P (Å)	2.69	3.06

^a^Published in reference [14]. ^b^Angle sum at phosphorous atom (pyramidality). ^c^Fenchyl–aryl dihedral angles (FAA, C1–C2–C3–O1) on the lone-pair side of phosphorus (FAA–lp) and at the substituent side (FAA) biaryl axis.

Moreover, the shorter P–O distance (2.4 Å) in O–BIFOP–Cl (**3**) and the nearly linear (176.5°) O–P–Cl arrangement (Scheme 4) suggest a neighbor-group effect through an O-lp donor to σ*P–Cl acceptor interaction, supporting chloride substitution (Figure 5, Scheme 4). Hypervalent P(III)–O interactions with similar P–O distances are documented for five membered rings [47–48] as well as for acyclic systems [49].

The computational analysis of the hydrolysis of the chlorophosphites BIFOP–Cl (**1**) and O–BIFOP–Cl (**3**, as well as the smaller model system 2-chloro-1,3,2-dioxaphospholane **8**) provides further comparison of the >P–Cl reactivity. The nucleophilic substitution reaction takes place at a triple-coordinated chlorophosphite (in R_2_PCl) due to a single-well potential energy surface [50–51]. The initial step of the water addition proceeds through the formation of the transition state (TS1) in which the oxygen atom of the water molecule binds to the phosphorous atom (Scheme 3, Table 2) and chloride substitution forms the product (G2). Here, chloride is replaced at the phosphorus center with the hydroxide nucleophile (Table 2).

**Table 2:** Computed relative energies (*E*_rel_, kcal/mol) for the reaction of **2**, **4** or **8** with water.

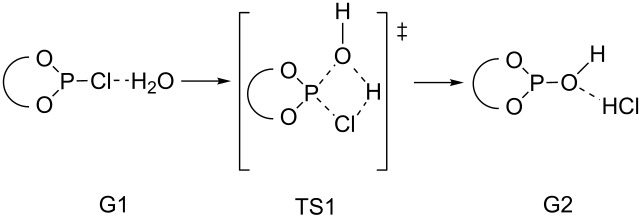				

Entry	Chlorophosphane	*E*_rel_ (G1)	*E*_rel_ (TS1)	*E*_rel_ (G2)

1^a^	BIFOP–Cl (**1**)	0.0	31.2	−4.7
2^a^	O–BIFOP–Cl (**3**)	0.0	22.5	−3.1
3^b^	[CH_2_O]_2_P–Cl (**8**)	0.0	18.3	−5.3

^a^BP86/SVP + ZPE. ^b^MP2/cc-p-VQZ//BP86/SVP + ZPE.

The relatively high hydrolyzation barrier of BIFOP–Cl (**1**, 31.2 kcal/mol) in comparison to O–BIFOP–Cl (**3**, 22.5 kcal/mol) and the smaller, glycol-based, chlorophosphite [CH_2_O]_2_P–Cl (**8**, 18.3 kcal/mol, Table 2) agrees with the experimental finding that BIFOP–Cl (**1**) is unusually robust against hydrolysis (Figure 1 and Figure 3). The lower hydrolysis barriers of **3** and **8** agree with the expected high reactivity of the >P–Cl in water [52–57].

**Figure 6:**
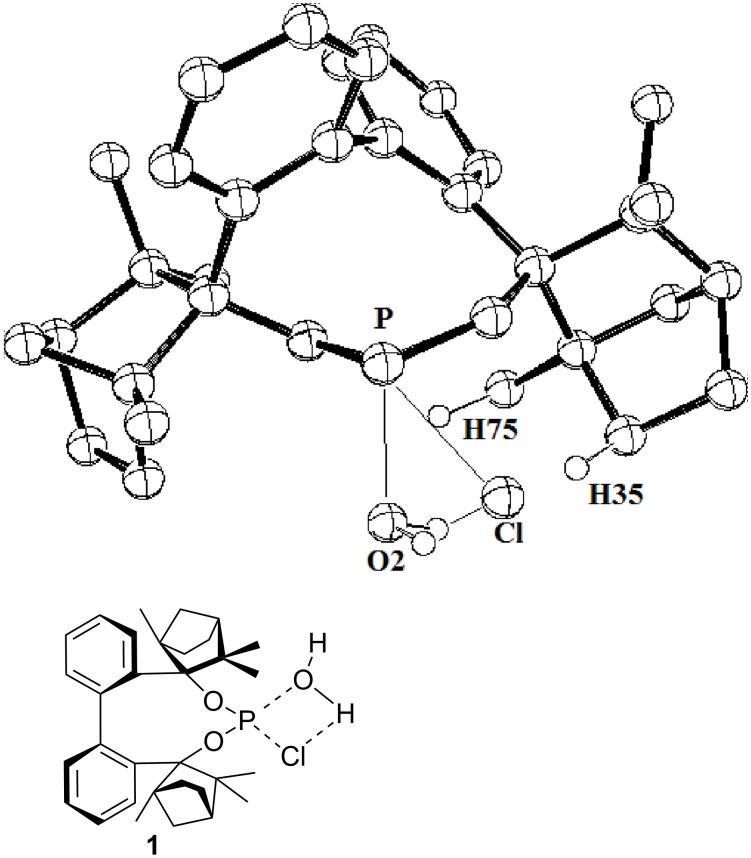
Transition state structure for the reaction of BIFOP–Cl (**1**) with water (BP86/def-SV(P)).

**Figure 7:**
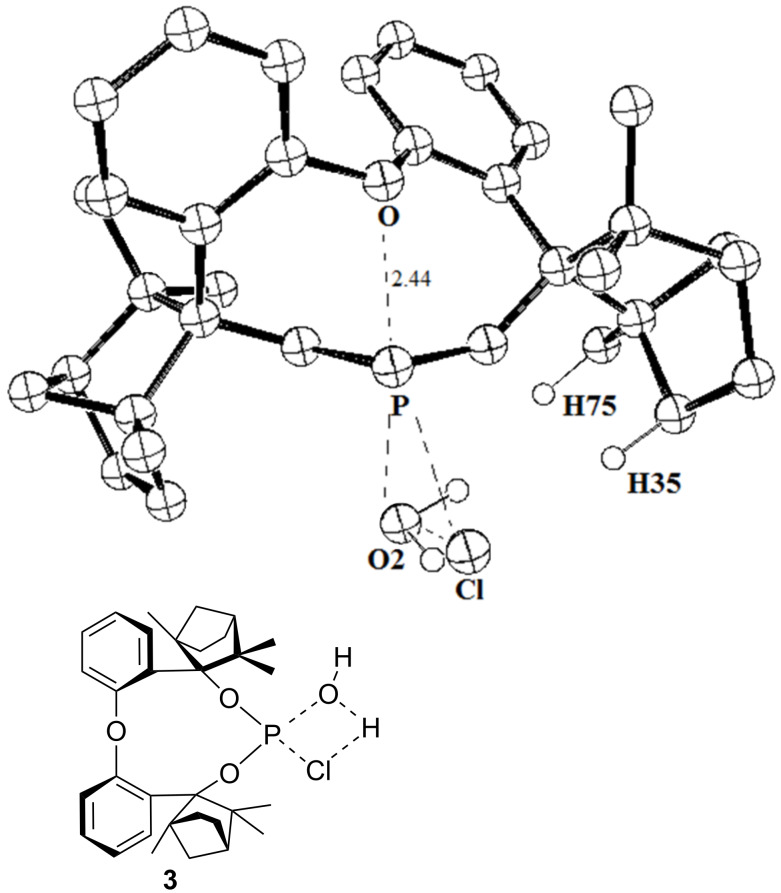
Transition state structure for the reaction of O-BIFOP–Cl (**3**) with water (BP86/def-SV(P)).

A comparison of the transition state structures of chlorophosphites **1** (Figure 6) and **3** (Figure 7) reveals a higher steric congestion of the P–Cl unit by the fenchane moiety in BIFOP–Cl (**1**) relative to O–BIFOP–Cl (**3**). In BIFOP–Cl (**1**), the shorter distances of the *endo*-oriented hydrogen atoms of the fenchane moiety (H35 and H75) to the Cl atom of the P–Cl unit and to the O atom of water (Table 3) prevent both the elimination of the chloride nucleofuge and the attack of the water nucleophile. This steric congestion of the transition state structures in reactions with water explains the surprisingly low reactivity of BIFOP–Cl (**1**, Figure 6) relative to the much more reactive O–BIFOP–Cl (**3**, Figure 7).

**Table 3:** Selected, computed distances in the transition state structures for the addition of water to chlorophosphites **1** and **3**.^a^

Distance	BIFOP–Cl (**1**)	O–BIFOP–Cl (**3**)

Cl–H35 (Å)	2.65	3.05
Cl–H75 (Å)	4.70	5.21
O2–H35 (Å)	2.68	2.98
O2–H75 (Å)	2.60	3.36

^a^BP86/def-SV(P) optimized transition state structures, cf. Figure 6 and Figure 7.

## Conclusion

Two fenchole-based chlorophosphites, BIFOP–Cl (**1**) and O–BIFOP–Cl (**3**), were studied with respect to their striking differences in regards to their reaction with water. While BIFOP–Cl (**1**) exhibits a surprisingly high stability against hydrolysis, O–BIFOP–Cl (**3**) reacts instantly with water, leading to cyclofenchene **6**. X-ray studies revealed that the increased reactivity of the intermediate carbenium ion and cyclopropane formation is due to a steric effect caused by the shielding of the fenchane groups and a hypervalent P(III)–O interaction. Formation of the cyclofenchene derivative **7** is explained by rearrangement via a 2-fenchyl carbocation. The DFT computations of the hydrolysis revealed a higher degree of steric congestion in BIFOP–Cl (**1**) caused by the fenchane units, relative to the less-shielded and hence much more reactive O–BIFOP–Cl (**3**). This result demonstrates that steric and electronic effects can be used to render the inherently highly reactive and electrophilic phosphorus–halogen units essentially inert against nucleophilic reagents. The stability of BIFOP–Cl (and other phosphorus–halogen systems) against nucleophiles promotes its application as a chiral ligand to be used in, for example, Pd catalysis [13–15].

## Experimental

All reactions were carried out under an inert argon atmosphere and in heated glassware using standard Schlenk techniques. Anhydrous solvents were obtained by distillation from sodium benzophenone ketyl. The NMR spectra were measured with Bruker instruments (Avance II 600, Avance II 300 and DPX Acance 300). Deuterated chloroform was used as solvent. The proton shifts are reported in ppm (δ) downfield from TMS and are referenced to residual signals of the solvent (CHCl_3_ 7.24 ppm for hydrogen, 77.0 ppm for carbon atoms). The coupling constants (*J*) are given in Hz. As an external standard, 85% phosphoric acid was used for the ^31^P NMR spectra. The infrared spectra were recorded on a Shimadzu, IRAffinity-1 instrument. The wavenumbers (ν) of the recorded IR signals are given in cm^−1^. The GC–MS spectra were recorded using an Agilent Technologies, Model GC 6890N gas chromatograph coupled with an HP 5973N series mass selective detector and an HP 7683 GC autosampler. Optical rotation was measured with an IBZ, Messtechnik POLAR LµP-WR polarimeter, using a 1 dm path length cell. The reactions were carried out under dry argon. X-ray analysis was performed with a Nonius, Kappa CCD diffractometer (Mo Kα, λ = 0.71073). The starting material, O–BIFOL, was obtained in an analogous manner to a procedure previously described [15].

### Diphenyl ether-2,2'-bisfencholphosphane chloride (O–BIFOP–Cl, **3**)

The O–BIFOP–Cl compound was prepared in a manner analogous to the procedure described in [15]. 1.48 mL (3 mmol) of *n*-butyllithium in hexane (1.6 M) was slowly added to a 200 mg solution (0.42 mmol) of O*–*BIFOL in 1.4 mL abs. THF at −20 °C. The mixture was stirred for 30 min at −20 °C, then for 1 h at rt. After again cooling to −20 °C, 0.06 mL (0.46 mmol) of freshly distilled PCl_3_ was slowly added and the reaction mixture was stirred for 6 h at rt. Recrystallization from Et_2_O/CH_2_Cl_2_ resulted in 111 mg (0.21 mmol, 50%) of compound **3** as colorless crystals. [α]_D_^20^ +46.47 (*c* 4.5, hexane); ^31^P NMR (125.5 MHz, CDCl_3_) δ 161.9; ^1^H NMR (300 MHz, CDCl_3_) δ 0.11 (s, 3H), 0.42 (s, 3H), 0.77 (s, 3H), 0.82 (s, 3H), 1.22–1.58 (m, 8H), 2.37 (d, *J* = 9 Hz, 1H), 2.49 (m, 3H), 2.45 (m, 3H), 2.76 (m, 4H), 6.75 (d, *J* = 6 Hz, 1H), 6.96 (t, *J* = 9 Hz, 1H), 7.17–7.22 (m, 2H), 7.54 (d, *J* = 6 Hz, 1H), 7.62 (d, *J* = 6 Hz, 1H); ^13^C NMR (75 MHz, CDCl_3_) δ 18.3, 21.1, 22.2, 22.7, 32.6, 38.6, 42.4, 42.9, 49.8, 49.4, 51.3, 52.7, 96.3, 115.2, 121.9, 122.8, 125.6, 128.4, 148.8; X-ray crystal data: C_32_H_41_O_4_P; *M*_r_ = 539.1 g·mol^−1^; space group: *P*2_1_2_1_2_1_; *a* = 12.2504(6), *b* = 14.9267(9), *c* = 30.6807(12) Å; *V* = 5610.2(5) Å^3^; *Z* = 8; ρ = 1.276 g·mL^−3^; *T* = 100(2) K; λ = 0.71073; μ = 0.123 mm^−1^; total reflections: 22204; unique reflections: 11385; observed: 5685 [I>2s(I)]; parameters refined: 679; R1 = 0.0611, wR2 = 0.0981; GOF = 0.924; H atoms bound to oxygen were refined, the positions of the H atoms bound to carbon were calculated.

### Diphenyl ether-2,2'-bisfencholphosphane hydride (O–BIFOP–H, **4**)

The O–BIFOP–H compound was prepared in a manner analogous to the procedure as described in [15]. 8.7 mg (0.23 mmol) of LiAlH_4_ was added to 100 mg (0.19 mmol) of O–BIFOP–Cl (**3**) in 1 mL of THF and the mixture was stirred for 3 h at rt. The solvent was removed in vacuum and the residue was taken up in 10 mL of toluene and stirred for 30 min at rt. After filtration through celite to remove LiCl and other salts, the resulting solution was concentrated in vacuum until precipitation. The recrystallization from toluene yielded 83 mg (0.16 mmol, 87%) of **4** as a white solid. [α]_D_^20^ +53.74 (*c* 2.8, hexane); ^31^P NMR (125.5 MHz, toluene-*d*_8_) δ 153.5 (^1^*J*_P–H_ = 190 Hz); ^1^H NMR (300 MHz, toluene-*d*_8_) δ 0.35 (s, 3H), 0.55 (s, 3H), 0.73 (s, 3H), 0.96 (t, *J* = 6 Hz, 1H), 1.04–1.11 (m, 6H), 1.22 (s, 3H), 1.31 (t, *J* = 6 Hz, 1H), 1.52 (d, *J* = 6 Hz, 2H), 1.68 (s, 1H), 1.72 (s, 1H), 6.62 (d, ^1^*J*_P–H_ = 190 Hz, 1H), 6.84–7.03 (m, 2H), 7.53 (d, *J* = 6 Hz, 1H); ^13^C NMR (75 MHz, toluene-*d*_8_) δ 18.3, 22.8, 23.8, 24.1, 24.8, 23.2, 34.4, 42.4, 43.5, 49.5, 52.4, 54.6, 97.9, 99.3, 116.9, 118.2, 122.5, 122.8, 123.9, 124.5, 125.0, 126.1, 136.8, 138.8, 145.2, 149.7.

### Diphenyl ether-2,2'-bisfencholphosphate (O-BIFOPH(O), **6**)

O_2_ was supplied to 83 mg (0.16 mmol) of O–BIFOP–H (**4**) for 5 min. The recrystallization from toluene yielded 38 mg (79%) of **6** as a colorless solid. [α]_D_^20^ +55.7 (*c* 4.5; hexane); ^31^P NMR (125.5 MHz, toluene-*d*_8_) δ −8.2 (^1^*J*_P–H_ = 710.8 Hz); ^1^H NMR (300 MHz, CDCl_3_) δ 0.41 (s, 3H), 0.52 (s, 3H), 0.57 (s, 3H), 0.82 (s, 3H), 1.04 (s, 1H), 1.20 (s, 1H), 1.28 (s, 6H), 1.34 (m, 6H), 1.37 (m, 6H), 1.49 (s, 4H), 1.65 (d, *J* = 9 Hz, 2H), 1.75 (m, 4H), 6.75 (d, ^1^*J*_P–H_ = 710.8 Hz, 1H), 6.96 (d, *J* = 6 Hz, 1H), 7.17 (t, *J* = 9 Hz, 2H), 6.64 (d, *J* = 6 Hz, 1H), 7.71 (d, *J* = 6 Hz, 1H); ^13^C NMR (75 MHz, CDCl_3_) δ 18.03, 18.17, 21.90, 22.97, 23.63, 23.73, 28.62, 29.69, 34.31, 35.60, 41.02, 42.33, 43.06, 48.73, 49.04, 49.31, 50.30, 55.60, 118.05, 119.87, 121.08, 121.69, 122.93, 123.36, 126.58, 127.82, 128.20, 129.97, 130.55, 131.46.

### Diphenyl ether-2,2’-biscyclofenchene-1,3,3-trimethyltricyclo[2.2.1.0]heptane (**7**)

2.9 mL (0.16 mmol) of H_2_O was slowly added to 83 mg of O–BIFOP–Cl (**3**, 0.16 mmol) in 2 mL of THF, and the mixture was stirred for 20 min at rt. The solvent was removed in vacuum, and the residue was taken up in 10 mL of toluene and filtered through celite. The resulting solution was concentrated in vacuum until precipitation. The recrystallization from toluene yielded 44 mg (65%) of **7** as colorless crystals. ^1^H NMR (300 MHz, CDCl_3_) δ 0.74 (s, 1H), 0.90 (s, 3H), 0.94 (s, 3H), 1.07 (s, 1H), 1.12 (s, 3H), 1.24–1.29 (m, 3H), 1.46 (s, 1H), 1.53 (d, *J* = 10 Hz, 2H), 1.84 (d, *J* = 10 Hz, 2H), 6.89 (d, *J* = 6 Hz, 1H), 6.99 (t, *J* = 9 Hz, 1H), 7.09 (d, *J* = 6 Hz, 1H), 7.19 (d, *J* = 6 Hz, 1H); ^13^C NMR (75 MHz, CDCl_3_) δ 15.63, 15.90, 21.76, 22.23, 22.61, 25.86, 27.09, 32.72, 33.04, 35.68, 38.20, 38.33, 42.61, 42.72, 47.65, 47.81, 118.62, 120.29, 121.47, 121.86, 126.86, 127.40, 127.91, 134.34, 134.69; IR (KBr) ν: 3334 (s), 2987 (vs), 1503 (m), 1434 (m); ESIMS (%) *m*/*z*: [M]^+^ 438.3; Anal. calcd for C_32_H_38_O (438.3 g·mol^−1^): C, 87.62; H, 8.73; found: C, 87.60; H, 9.19. X-ray crystal data: C_32_H_38_O; *M*_r_ = 438.6 g·mol^−1^; space group: *P*2_1_; *a* = 7.2593(6), *b* = 16.903(2), *c* = 20.472(2) Å; *V* = 2508.4(4) Å^3^; *Z* = 4; ρ = 1.161 g·mL^−3^; *T* = 100(2) K; λ = 0.71073; μ = 0.068 mm^−1^; total reflections: 10449; unique reflections: 8172; observed: 3859 [I>2s(I)]; parameters refined: 578; R1 = 0.1649, wR2 = 0.3899; GOF = 1.264; H atoms bound to oxygen were refined, the positions of the H atoms bound to carbon were calculated.

### Computational details

The computations were performed with the program package TURBOMOLE-5.10 [58–60]. The employed functional was BP86 with an m3 grid size combined with the contracted, SVP basis set from Ahlrichs et al. The resolution-of-identity approximation for a two-electron integral evaluation was used. All stationary points were fully optimized and confirmed by separate analytical frequency calculations. The transition state structures were optimized with quasi-Newton–Raphson methods by using the Powell update algorithm for Hessian matrix approximation (analytical frequency calculation subsequent). The absolute energies were zero-point-corrected with the vibrational information resulting from the harmonic analytical frequency calculations.
